# Therapeutic advances with KRAS^G12C^ inhibitors and combination strategies in non-small cell lung cancer brain metastases

**DOI:** 10.1038/s41417-026-01003-0

**Published:** 2026-02-09

**Authors:** Debanjan Bhattacharya, Benjamin Roman, Sanjana Reddy

**Affiliations:** Department of Neurology and Rehabilitation Medicine, University of Cincinnati College of Medicine, Cincinnati, OH USA

**Keywords:** CNS cancer, Cancer therapy, Non-small-cell lung cancer

## Abstract

Non-small cell lung cancer (NSCLC) frequently metastasizes to the brain in approximately 20–40% of cases. Mutations in the Kirsten rat sarcoma viral oncogene homologue (KRAS) are common in NSCLC, with the KRAS^G12C^ variant accounting for approximately 40% of KRAS-mutant cases. Up to 40% of NSCLC patients harboring the KRAS^G12C^ mutation develop brain metastases during follow-up, and a substantial proportion present with brain metastases at diagnosis. While KRAS^G12C^ inhibitors such as sotorasib and adagrasib are approved therapies, most patients with KRAS^G12C^ mutant NSCLC experience disease progression within 5 to 6 months. Emerging KRAS^G12C^inhibitors, such as adagrasib, RMC-6236, and olomorasib, show intracranial activity in KRAS^G12C^ mutant NSCLC brain metastases, but adaptive resistance limits their effectiveness as monotherapies. This article examines the clinical and translational application of specific next-generation blood-brain barrier penetrant KRAS^G12C^ inhibitors, such as sotorasib, adagrasib, olomorasib, RMC-6236, and D3S-001, and their rational integration with radiation therapy, targeted therapies, and immunotherapies to overcome therapeutic resistance in patients with NSCLC brain metastases. This review summarizes recent advances aimed at enhancing intracranial tumor control and overall survival in patients with NSCLC brain metastases through the use of next-generation KRAS^G12C^ inhibitors and multimodal therapies.

## Introduction

Lung cancer is the most common type of cancer in the United States and has the highest mortality rate, with an expected 120,000 deaths in 2025 [1]. This is 2.5 times the deaths from colorectal and pancreatic cancer combined. Non-small cell lung cancer (NSCLC) is the most prevalent, accounting for 80–85% of lung cancer cases [1]. NSCLC has three main histological types: adenocarcinoma, squamous cell carcinoma, and large cell carcinoma. NSCLC metastasizes to the brain in 30–40% of patients [2]. NSCLC patients with brain metastasis face a grim prognosis. Although cranial radiotherapy in the form of either whole brain radiation therapy (WBRT) or stereotactic radiosurgery (SRS) is a part of a multimodal treatment for NSCLC brain metastasis, the median survival is only about 7–10 months [3, 4]. Management of brain metastasis represents a challenge for conventional therapies due to the resilient nature of the metastatic brain tumors [4]. In approximately 30% of cases, mutations in the rat sarcoma virus (RAS) gene drive a particularly aggressive form of NSCLC, a phenomenon also observed in other cancers. RAS codes for a family of small GTPase proteins, which are involved in transmitting signals within cells and play a critical role in cellular processes [5]. RAS has a GDP-bound inactive form and a GTP-bound active form. Upon activation of a transmembrane receptor, the α subunit of RAS binds guanine triphosphate (GTP) [RAS(ON] and exerts downstream effects, including activating signaling pathways for cellular processes [5]. When guanine diphosphate GDP is bound, the RAS becomes inactive, RAS(OFF), and the downstream signaling pathways are not activated [5]. Mediation between the RAS(ON) and RAS(OFF) states is accomplished by a guanine nucleotide exchange factor (GEF). Kirsten rat sarcoma viral oncogene homologue (KRAS) is a RAS isoform frequently mutated in NSCLC, and the substitution of glycine 12 with cysteine (G12C) is a common KRAS mutation [6]. Missense mutations in KRAS cause alterations to the switch-II region of the molecule, which impairs the effector molecule (GAP) binding and causes an elevated percentage of active GTP-bound KRAS [7]. The most common KRAS mutation is the amino acid substitution of cysteine for glycine in codon 12, leading to the KRAS^G12C^ mutant [8, 9]. KRAS^G12C^ accounts for approximately 40% of KRAS mutations in NSCLC, followed by KRAS^G12V^ (∼18–21%), and KRAS^G12D^ (∼17–18%) substitutions [10, 11]. KRAS codon 12 mutations most commonly result in G12C, G12V, or G12D substitutions, which account for 40%, 19% and 15% of KRAS mutations in NSCLCs, respectively [12]. Mutations in the KRAS gene impair its intrinsic GTPase activity, resulting in constitutive activation of the protein and uncontrolled cell proliferation.

Brain metastases are highly prevalent in patients with NSCLC harboring KRAS^G12C^ mutations, with about 28% of patients having detectable brain metastases at diagnosis. The incidence of brain metastasis in KRASG12C-positive NSCLC patients increases to 40% at follow-up [13].

While previously considered undruggable, new therapies have emerged that target KRAS(OFF) and KRAS(ON) proteins. KRAS^G12C^ mutations are targetable, and researchers have developed preclinical models of KRAS^G12C^ mutant brain metastasis to assess the efficacy of available brain-penetrant KRAS^G12C^-targeted agents in NSCLC brain metastasis.

The blood-brain barrier (BBB) shields the brain from harmful substances while permitting essential nutrients to pass, but it also blocks many drugs, reducing their effectiveness. Therefore, while systemic chemotherapy and targeted therapies may be effective for the treatment of primary tumors, the treatment of brain metastases is more challenging. However, in patients with stage IV NSCLC, both KRASG12C and KRAS non-G12C mutations confer a similar likelihood of brain metastasis [14, 15].

## KRAS^G12C^ mutations and development of brain metastasis in NSCLC

About 40% of NSCLC patients harboring the KRAS^G12C^ mutation ultimately develop brain metastases that lead to poor clinical outcomes [16]. KRAS^G12C^ in NSCLC is more frequently observed in Caucasian and African American patients compared to Asian patients [17]. KRAS mutations, including KRAS^G12C^, are important prognostic markers in patients with NSCLC, as they affect overall survival and the risk of distant brain metastasis. Distant brain failure indicates the development of non-contiguous brain metastases outside the area treated with initial stereotaxic radiosurgery (SRS), indicating a failure of stereotactic radiosurgery (SRS) treatment to control brain metastases [18]. In patients with NSCLC and brain metastases, the presence of KRAS mutations is associated with a higher incidence of distant brain failure [18]. In a recent retrospective cohort study of 374 patients with KRAS-mutated metastatic NSCLC (40% KRAS^G12C^ and 60% non-G12C), approximately 90% developed brain metastases during their disease course, with nearly half presenting with intracranial disease within 12 months of metastatic diagnosis [15].

The glycine-to-cysteine substitution mutation at position 12 (G12C) of the KRAS protein interferes with guanosine triphosphate (GTP) hydrolysis, thus keeping KRAS primarily in the active, GTP-bound state [7]. This disrupts the everyday cycling between the inactive GDP-bound state and an active GTP-bound state. The transition is regulated by guanine nucleotide exchange factors (GEFs) and GTPase-activating proteins (GAPs). However, the KRAS^G12C^ mutation impairs this regulation, leading to the accumulation of the active KRAS^G12C^-GTP protein [7, 19]. When KRAS^G12C^ is in this active state, it triggers activation of key signaling pathways, including the RAF/MEK/ERK and PI3K/AKT/mTOR pathways [20]. In the RAF/MEK/ERK pathway, the active KRAS-GTP interacts with RAF kinases, leading to their dimerization and activation. This RAF activation triggers a phosphorylation cascade that activates MEK, which in turn activates ERK and promotes cellular proliferation [20]. The activated KRAS^G12C^-GTP protein promotes recruitment of PI3K, leading to activation (phosphorylation) of AKT, which then phosphorylates and inactivates pro-apoptotic factors like BAD and Caspase-9 and also activates anti-apoptotic factors like Bcl2, allowing the cells to survive under conditions of stress [7, 20]. Activated form of KRAS-GTP activates PI3K/AKT signaling, which subsequently activates mTOR. Activated mTOR signaling promotes cancer cell growth by enhancing protein synthesis through increased ribosome production, stimulating lipid biosynthesis [21]. Another key mechanism by which the KRAS^G12C^ mutant protein promotes metastasis is by remodeling the tumor microenvironment (TME) [22]. KRAS mutations regulate the TME by interacting with proteins involved in angiogenesis, such as integrin β-4, to promote blood vessel formation, thereby increasing invasion [22]. KRAS mutations also contribute to an immunosuppressive TME by limiting T cell infiltration and recruiting immune suppressive cells [23].

Some KRAS^G12C^ inhibitors have been approved for monotherapy in patients with advanced KRAS^G12C^ mutant NSCLC. While brain-penetrant targeted therapies for other oncogenic drivers have largely replaced radiation treatment and surgery in the treatment of active brain metastases, comparable targeted therapies for KRAS^G12C^ mutant NSCLC have only recently emerged [24]. Although some KRAS^G12C^ inhibitors, such as sotorasib (AMG-510) and adagrasib (MRTX849), have received federal regulatory approval as monotherapies for patients with NSCLC harboring a KRAS^G12C^ mutation, most patients experience disease progression within 5–6 months [25–27]. This review highlights emerging strategies for treating brain metastases in KRAS^G12C^mutant NSCLC patients, emphasizing combination therapies involving KRAS^G12C^ inhibitors, targeted therapies, immunotherapies, and radiation. It also discusses ongoing clinical trials investigating these combinations and their effects on intracranial disease control and patient outcomes with targeted therapies, immunotherapies, and radiation. Although this review focuses on KRAS^G12C^ mutant NSCLC, the direct inhibitors and their combination therapies discussed here are potentially applicable to other KRAS^G12C^ mutant solid cancers.

## Effect of KRAS co-mutations on brain metastasis occurrence and outcome

Secondary mutations can synergize with KRAS mutations to enhance tumor cell invasiveness and migration capacity. In KRAS^G12C^ mutant NSCLC, the presence of co-occurring mutations in tumor-suppressing genes, such as Kelch-like ECH-associated protein 1 (KEAP1) and cyclin-dependent kinase inhibitor 2 A (CDKN2A), is associated with shorter progression-free survival (PFS), reduced overall survival (OS), and rapid disease progression [28]. KEAP1 encodes the KEAP1 protein, which regulates the activity of nuclear factor erythroid 2-related factor 2 (Nrf2), a transcription factor involved in redox homeostasis [29]. Nrf2 overexpression is associated with increased tumor aggressiveness in KRAS-mutant NSCLC at later stages, but acts as a tumor suppressor in early-stage disease [30]. KEAP1 mutations also may occur in tandem with the loss or inactivation of liver kinase B1 (LKB1), encoded by serine/threonine kinase 11 (STK11) [28]. Concurrent KRAS/LKB1 mutations are associated with increased brain metastasis compared with other mutations, but LKB1 mutations alone are not associated with tumorigenesis [31]. Copy number mutations in mutant KRAS/LKB1 are associated with a higher incidence of brain metastasis versus KRAS^WT^ or LKB1^WT^ [32].

SMARCA4 encodes the chromatin remodeling protein Brahma-Related Gene 1 (BRG1), and mutations in SMARCA4 in NSCLC are associated with poor outcomes [33]. The prognosis becomes significantly unfavorable when SMARCA4 mutations co-occur with KRAS^G12C^ mutation in primary NSCLC patients [28]. Additionally, mutations and deletions in the CDKN2A and CDKN2B (CDKN2A/B) are frequently observed during the progression of NSCLC to brain metastases [34]. CDKN2A/B mutations are reported to be more prevalent in lung adenocarcinoma brain tumor metastases compared to the primary tumor samples from patients [35]. Alterations in CDKN2A/B are also poor predictors of intracranial disease progression [35]. Mutations in CDKN2A/B frequently co-occur with KRAS mutations, and the simultaneous presence of both alterations synergistically enhances metastatic potential [36].

## Emerging KRAS^G12C^ inhibitors with therapeutic potential

KRAS^G12C^ inhibitors are broadly classified into two classes based on their mechanism of action. They are KRAS^G12C^(OFF) inhibitors that selectively bind to the inactive KRAS protein in its guanine diphosphate (GDP)-bound state. By stabilizing KRAS in this GDP-bound conformation, these inhibitors effectively keep KRAS “turned off.” The other category comprises KRAS (ON) inhibitors, which directly bind to the active guanine triphosphate (GTP)-bound KRAS protein. Mechanistically, KRAS (ON) inhibitors can be either covalent, highly selective inhibitors of KRAS^G12C^ (ON) proteins or non-covalent, multi-selective inhibitors with potent activity against both wild-type and mutant KRAS (ON) proteins. The KRAS^G12D^ protein has lower intrinsic GTPase activity than KRAS^G12C^; so most KRAS^G12D^ proteins will be GTP-bound. Thus, agents that target KRAS^G12D^-GTP, as well as other RAS proteins, exploit the RAS activation cycle in which guanine diphosphate (GDP) bound, RAS adopts an inactive RAS(OFF) conformtion. In contrast, KRAS^G12C^ (OFF) inhibitors bind the inactive, GDP-bound form of the KRAS^G12C^ mutant protein and prevent its reactivation. They act more like “OFF” state stabilizers for that specific KRAS mutant protein.

Some of the major KRAS(OFF) inhibitors are sotorasib, adagrasib, olomorasib (LY3537982), divarasib (GDC-6036), MK-1084, garsorasib (D-1553), fulzerasib (IBI351/GFH925), opnurasib (JDQ-443), glecirasib (JAB-21822) (G12C), and [9, 37, 38]. Some of the important KRAS(ON) inhibitors evaluated in preclinical studies are RMC-6236, RMC-6291 and BBO-8520 [9]. Table 1 lists prominent KRAS^G12C^ specific inhibitors, including both KRAS(OFF) inhibitors that target the inactive GDP-bound form and KRAS(ON) inhibitors that target the active GTP-bound form. An alternative approach to target mutant KRAS^G12C^ protein involves cyclophilin A recruitment and uses molecular glue compounds that promote a complex between KRAS^G12C^ and cyclophilin A (CyPA). The cyclophilin-A (CyPA) recruiter RMC-6291 forms an inhibitory tri-complex with KRAS^G12C^-GTP and CyPA, sterically blocking effector activation such as RAF. Unlike sotorasib or adagrasib, which trap KRAS in its inactive state, RMC-6291 binds the active KRAS–GTP form into an inhibitory tri-complex with cyclophilin-A. In xenograft models, this agent showed better tumor reduction compared than adagrasib, prompting ongoing clinical trials. A related compound, RMC-6236, a pan-KRAS cycliphilin-A (CyPA) recruiter that acts as a pan-KRAS inhibitor, is also under clinical evaluation[39]. Here, we highlight several of the most prominent KRAS^G12C^ inhibitors. It should be noted that some of the data discussed in this review are derived from studies in the broader NSCLC population, rather than specifically from patients with brain metastases.

**Table 1:** List of some KRAS^G12C^ targeting inhibitors under development in NSCLC [9].

Drug	Mechanism
Sotorasib (AMG510) [9]	G12C OFF (GDP-bound)
Adagrasib (MRTX849) [9, 37]	G12C OFF (GDP-bound)
Divarasib (GDC-6036) [38]	G12C OFF (GDP-bound)
Olomorasib (LY3537982) [45]	G12C OFF (GDP-bound)
MK-1084 [90]	G12C OFF (GDP-bound)
D3S-001[64]	G12C OFF (GDP-bound)
Garsorasib (D-1553) [91]	G12C OFF (GDP-bound)
Fulzerasib (IBI351/GFH925) [49]	G12C OFF (GDP-bound)
Glecirasib (JAB-21822) [92]	G12C OFF (GDP-bound)
RMC-6236 [39]	G12C ON (GTP-bound)
RMC-6291 [93]	G12C ON (GTP-bound)

### Sotorasib

Sotorasib (formerly known as AMG 510) is an FDA-approved drug that targets the inactive GDP-bound form of the KRAS^G12C^ protein [37, 40]. Sotorasib received accelerated approval from the FDA in May 2021 based on the CodeBreak 100 phase II trial on sotorasib monotherapy. Becoming the first approved KRAS^G12C^ inhibitor to treat KRASG12C-mutated NSCLC patients who had failed at least one prior therapy [25, 41]. Current compounds are in clinical trials for the treatment of non-small cell lung cancer (NSCLC). A list of KRAS targeting compounds and their mechanisms of action is summarized in Table 2. In a clinical trial with sotorasib, the KRAS^G12C^ inhibitor caused complete tumor regression in 4 patients (3.2%), partial responses in 42 patients (33.9%), and stable disease in 54 patients (43.5%) [25]. Notably, sotorasib elicited responses in patients with co-mutations. In KRAS^G12C^/LKB1^MUT^/KEAP1^WT^ NSCLC, 50% of lung cancer patients responded to treatment [25]. Patients with KRAS^G12C^/LKB1^MUT^/KEAP1^MUT^ tumors had a response rate of 23%, and those with KRAS^G12C^/LKB1^WT^/KEAP1^MUT^ tumors had a response rate of 14% [25]. Lebouille-Veldman et al. evaluated the efficacy of sotorasib in patients with active brain metastases in a retrospective study. Patients with active brain metastasis were treated either with sotorasib monotherapy or in combination with localized therapies such as radiation or surgery. The results were compared with those of patients with stable brain metastasis treated with Sotorasib. Results showed that 57% of patients (*n* = 17/30) on sotorasib experienced intracranial disease progression, including 44% (*n* = 8/15) of patients with previously stable brain metastasis that also progressed on sotorasib alone [42]. Overall survival (OS) for the three groups was still dismal, as patients with active brain metastasis treated with sotorasib alone had an average OS of only 4 months [42]. Patients with active brain metastases receiving sotorasib plus a localized therapy had an average overall survival (OS) of 6 months. In contrast, those with stable brain metastases treated with sotorasib alone had an average OS of 12 months [42]. Clearly, the clinical efficacy of sotorasib depends on patient profiling, and patients with stable brain metastasis who receive standard-of-care treatment have a better outcome. Targeted therapies like sotorasib underscore the importance of molecular profiling in advanced non-squamous NSCLC, as they significantly improve outcomes for patients with driver mutations. Although sotorasib has shown benefits in patients with previously stable brain metastasis from lung cancer, a multidisciplinary approach is essential for post-treatment evaluation. A phase-2 clinical trial of single-agent KRAS^G12C^ inhibitors in lung cancer patients showed that sotorasib provides clinical benefit; however, adaptive resistance develops more rapidly than with other targeted agents [25]. Furthermore, a phase 3 trial revealed additional challenges with sotorasib treatment. The CodeBreak 200 trial (NCT04303780), an open-label randomized phase 3 trial, demonstrated that the median progression-free survival was significantly higher (5.6 months) for 169 patients treated with sotorasib (AMG510) compared to 4.5 months for 174 patients treated with docetaxel [26]. Sotorasib also showed a more favorable safety profile compared to docetaxel [26]. Patient-reported outcomes favored sotorasib and indicated a better quality of life for patients on this treatment. Median overall survival (OS) did not differ significantly between these groups. However, sotorasib as monotherapy for advanced NSCLC patients, prolonged progression-free survival compared to those on docetaxel.

**Table 2:** Clinical trials of some prominent KRAS inhibitors reported in NSCLC.

Drug	Clinical trial	Phase	BBB Permeable	Inclusion criteria	Exclusion criteria	MOA	Results	Ref
Sotorasib (AMG 510)	CodeBreak 100	1/2	Some permeability	Locally advanced or metastatic non-small cell lung cancer (NSCLC) with confirmed KRAS^G12C^ mutation. Disease progression following prior platinum-based chemotherapy or anti-PD-1/PD-L1 immunotherapy	Active brain metastasis, although patients with previously treated brain metastasis were eligible. Systemic anticancer therapy within 28 days or therapeutic/palliative radiation therapy within 2 weeks before starting the study drug	Covalently binds to cysteine-12 in mutated KRAS, trapping it in an inactive state, inhibiting KRAS oncogenic signaling.	ORR: 41% (95% CI, 33.3–48.4%) mPFS: 6.3 mo (95% CI, 5.3 to 8.2 mo) mOS: 12.5 mo (95% CI, 10.0 to 17.8 mo)	[94, 95]
Adagrasib (MRTX849)	KRYSTAL-1	1/2	Yes, high BBB permeability.	Adults (≥ 18 years) with histologically confirmed unresectable or metastatic NSCLC harboring a KRAS^G12C^ mutation. For NSCLC cohorts: patients with locally advanced or metastatic NSCLC who have received at least one prior systemic therapy, including platinum-based chemotherapy and immune checkpoint inhibition. Patients with stable brain metastases were included if adequately treated and neurologically stable before enrollment.	Active brain metastases or leptomeningeal carcinomatosis Presence of other active cancer. A history of intestinal disease or major gastric surgery that can alter the absorption of oral medications or impair the ability to swallow them.	It covalently and irreversibly binds to KRAS^G12C^, locking in the inactive GDP-bound state.	ORR: 42.9% (95% CI, 33.5–52.6%) mPFS: 6.5 mo (95% CI, 4.7 to 8.4 mo) mOS: 11.7 mo (95% CI, 9.2 mo to NE)	[96]
Daraxonrasib (RMC-6236)	RASolve 301) (NCT06881784). To assess if daraxonrasib treatment will improve PFS or overall survival compared to docetaxel chemotherapy in NSCLC patients.	3	Yes. High permeability	Adults (≥ 18 years) with pathologically confirmed NSCLC, either locally advanced or metastatic, not controlled by curative surgery or radiotherapy. Eastern Cooperative Oncology Group (ECOG) performance status of 0 or 1.	Prior treatment with direct RAS-targeted therapy or docetaxel. Untreated CNS metastases. Ongoing anti-cancer therapy. Significant comorbidities like cardiovascular disease, lung disease, or impaired GI function.	Binds cyclophilin A (CypA), remodeling the CypA surface to create a complex with high affinity for the GTP-bound state of mutant & wild-type KRAS variants. Noncovalent CypA:compound: KRAS tri-complex blocks KRAS-effector interactions.	Results are not yet available as of 2025	NCT06881784[39]
RMC-7977	Pre-clinical and early clinical stages	Not yet in clinical trial	High permeability	Not yet in clinical trial		Inhibits RAS pathway signaling by forming RMC-7977–CyPA–KRAS-GTP tricomplex with cyclophilin A (CyPA) and RAS-GTP.		[39, 97, 98]
D3S-001	NCT05410145	1	Yes	Histologically or cytologically confirmed metastatic or locally advanced solid tumor with documented KRAS p.G12C mutation (identified within the past 5 years from tumor tissue or blood). ECOG performance status of 0 or 1.	Uncontrolled illness, including but not limited to ongoing or active infection, uncontrolled or significant cardiovascular disease. The subject has unresolved treatment-related toxicities from previous anticancer therapy.	Rapidly and irreversibly binds to the Cys12 of KRAS^G12C^, locking the protein in the inactive state.	Recruiting patients as of June 2025	[64, 99]
Olomorasib plus Pembrolizumab vs Placebo plus Pembrolizumab in those patients with PD-L1 expression ≥50%	SUNRAY-01	3	Yes	Adults (≥ 18 years) with histologically confirmed NSCLC with Stage IIIB-IIIC or Stage IV disease, not suitable for curative radical surgery or radiation therapy. Disease with evidence of KRAS G12C mutation. Must have known PD-L1 expression Part A: Greater than or equal to (≥)50 percent (%). Part B: 0% to 100%. ECOG performance status of 0 or 1. Estimated life expectancy ≥12 weeks.	Validated oncogenic driver mutation or alteration in genes such as EGFR, ALK, HER2, or ROS1. Presence of known CNS metastasis or carcinomatous meningitis. Prior systemic therapy (chemotherapy, immunotherapy, targeted therapy, or biological therapy) for advanced or metastatic NSCLC.	Covalent inhibitor of the GDP-bound form of KRAS^G12C^ as it binds to it.	Ongoing (no results available at the time of the manuscript in 2025)	
Olomorasib + pembrolizumab (part A) Olomorasib in combination with durvalumab (part B)	SUNRAY-02	3	Yes. Recent Studies indicated intracranial activity in KRAS^G12C^-mutated NSCLC brain metastasis	Part A: Clinical Stage II-IIIB NSCLC treated with presurgical chemoimmunotherapy with residual tumor present at the time of surgery Pathologic Stage II-IIIB (N2) NSCLC treated with initial upfront resection. Part B: Clinical Stage III, unresectable NSCLC, without progression on concurrent platinum-based chemoradiotherapy. NSCLC with evidence of KRASG12C mutation. Must have PD-L1 expression.	Have known changes in the EGFR or ALK genes. Had any immune-related side effect or allergic reaction (Grade 3 or higher) from a previous immunotherapy. Presence of another cancer that is progressing or requires active treatment within the past 3 years.	Covalent inhibitor of the GDP-bound form of KRAS^G12C^ as it binds to it.	Ongoing (no results posted at the time of this manuscript)	[9, 45]
Divarasib (GDC-6036)	NCT04449874	1	Yes	For NSCL cohort: Histologically documented advanced or metastatic NSCLC with KRAS G12C mutation.	Active brain metastases. Clinically significant cardiovascular dysfunction or liver failure. Malabsorption or any other condition that interferes enteral absorption.		ORR: 53.4% (95% CI: 39.9–66.7%) mPFS: 13.1 mo (95% CI, 8.8 mo to NE)	[62]
MK1084 monotherapy (Arm 1) ML1084 plus Pembrolizumab (Arm 2) MK-1084 plus Pembrolizumab plus chemotherapy (carboplatin and pemetrexed) (Arm 3)	KANDLELIT-001	1	Not yet reported	For Arm 1: Has locally advanced unresectable or metastatic NSCLC with histologically OR blood-based confirmation of KRAS^G12C^ mutation who has received at least 1 line of therapy for the systemic disease. For Arm 2: Untreated metastatic non-small cell lung cancer (NSCLC) with histologically OR blood-based confirmation of KRAS^G12C^ mutation and histologic confirmation of programmed cell death ligand 1 (PD-L1) tumor proportion score (TPS) ≥ 1%	Prior second malignancy, unless curative treatment has been completed and no evidence of malignancy for 5 years. Has received chemotherapy, definitive radiation, or biological cancer therapy within 4 weeks (2 weeks for palliative radiation) before first dose of study intervention. Has an autoimmune disease requiring systemic therapy.	Covalent inhibitor of the GDP-bound form of KRAS^G12C^ as	ORR: 38% when used as monotherapy. (*n* = 21) ORR: 77% When MK1084 used in combination with Pembrolizumab (*n* = 69) ORR: 53% MK1084 + Pembrolizumab + chemotherapy (carboplatin and pemetrexed) (*n* = 40)	

A post hoc analysis of CodeBreaK 100 showed that 16 (88%) patients achieved intracranial disease control, and two patients achieved complete response (CR) following Sototasib treatment [43]. Overall, intracranial disease control was achieved in 14 of 16 patients (87.5%) with evaluable BM. Safety in the BM group was consistent with previous reports [43].

### Adagrasib

MRTX849, also known as Adagrasib, is the second KRAS^G12C^ small-molecule inhibitor that binds to the inactive (GDP-bound) form of the KRASG12C protein. Adagrasib received accelerated approval from the FDA in December 2022 for use in previously treated advanced KRAS^G12C^ mutant NSCLC based on the KRYSTAL-1 trial, which reported a 42.9% objective response rate (ORR) (48 out of 112 patients), with a median PFS of 6.5 months and OS of 12.6 months [27]. Adagrasib is still in clinical trials (phase 2) for locally advanced and metastatic KRAS^G12C^ NSCLC (NCT03785249). Adagrasib is also in a Phase 3 trial (KRYSTAL-12: Phase 3 Adagrasib vs Docetaxel, NCT04685135). Preclinical data strongly suggest that adagrasib can penetrate the blood-brain barrier (BBB) in a dose-dependent manner and can accumulate in the brain tissue. In mouse models, Adagrasib significantly improved overall survival and lowered tumor volume compared to the control [16]. Notably, adagrasib has shown promising activity in treating NSCLC brain metastases, which affect approximately 40% of patients with KRAS-mutant NSCLC during their disease progression.

Adagrasib is the only KRAS^G12C^ inhibitor with data regarding activity in untreated brain metastases, with a central nervous system ORR of 42% in the KRYSTAL-1 trial [44]. The intracranial (IC) disease control rate (DCR) was 90% while the median IC progression-free survival (PFS) was 5.4 months (*n* = 19, 12-month PFS, 33.9%) in the KRYSTAL-1 trial [44]. The median IC duration of response (DOR) is 12.7 months.

In the KRYSTAL-1 trial, adagrasib demonstrated intracranial responses in patients with untreated or previously treated brain metastases. The intracranial Objective Response Rate (ORR) was ~33%, and the systemic disease control rate was greater than 80%. In clinical trials (*n* = 112), one patient (0.9%) taking adagrasib achieved a complete response, 47 (42.0%) had a partial response, and 41 (36.6%) had stable disease for at least 6 weeks [27]. Patients with brain metastases and some degree of disease control with conventional therapies were treated with adagrasib, as it demonstrated blood-brain barrier permeability [27]. Preclinical studies indicate that adagrasib penetrates cerebrospinal fluid (CSF) at clinically relevant levels, leading to tumor regression. It inhibits the P-glycoprotein multi-drug resistance (MDR) pump, which may facilitate its central nervous system (CNS) penetration. Additionally, adagrasib’s unbound brain-to-plasma partition coefficient, a measure of blood-brain barrier (BBB) permeability, is comparable to that of Osimertinib, known for intracranial activity [16]. Adapting the RANO-BM criteria, 33 of 42 patients with stable baseline brain metastasis (treated with radiation therapy) were radiographically evaluated, and these patients showed an intracranial (IC) objective response rate (ORR) of 33.3% [27]. In patients with previously untreated brain metastasis (*n* = 25), the intracranial ORR was 42% (95% CI, 20.3 to 66.5), with three patients achieving a complete response and five achieving a partial response [44]. The progression-free survival (PFS) for patients with untreated brain metastasis was 5.4 months with an OS of 11.4 months [44]. Upon stratification by co-mutation, the ORRs for concurrent STK11^MUT^ were 40.5%, KEAP1^MUT^ 28.6%, TP53 51.4%, and concurrent CDKN2A^MUT^ 58.3%, although responses were lower when multiple co-mutations occurred [27].

### Olomorasib

Olomorasib (LY3537982) is a selective, second-generation covalent inhibitor of GDP-bound KRAS^G12C^, with evidence of CNS activity [45]. It exerts antitumor activity as monotherapy and in combination with other anticancer therapies in preclinical models. Olomorasib (LY3537982), the investigational drug, showed dose-dependent tumor growth inhibition in vivo as a single agent in a KRAS^G12C^ NSCLC patient-derived xenograft (PDX) model [9, 45]. In September 2025, the USA Food and Drug Administration (FDA) granted the Breakthrough Therapy designation to olomorasib in combination with anti-PD-1 therapy pembrolizumab (immunotherapeutic agent) for the first-line treatment of unresectable advanced or metastatic NSCLC with KRAS^G12C^ mutation and PD-L1 expression ≥50%. This designation is based on the results from the Phase 1/2 LOXO-RAS-20001 (NCT04956640) trial and the dose optimization part of the Phase 3 SUNRAY-01 trial (NCT06890598). Olomorasib monotherapy has demonstrated consistent antitumor activity in KRASG12C mutant NSCLC. Among 39 NSCLC patients previously treated with a KRAS^G12C^ inhibitor, 63% of whom had received such therapy immediately before olomorasib, the objective response rate (ORR) was 41% with olomorasib. The median progression-free survival (PFS) was 8.1 months [46].

Due to its favorable pharmacokinetic properties, it has advanced to clinical testing in metastatic NSCLC. The clinical trial SUNRAY-02 is a phase 3, multicenter, double-masked, placebo-controlled study assessing the effect of Olomorasib in combination with standard-of-care immunotherapy (pembrolizumab) in participants with resected or unresectable KRAS^G12C^ mutant, NSCLC who have recovered from any previous treatment (Table 2). Patients with KRAS^G12C^ mutant, locally advanced or metastatic non-small cell lung cancer, comparing first-line treatment of olomorasib and pembrolizumab vs placebo and pembrolizumab in patients with PD-L1 expression ≥50% or olomorasib and pembrolizumab, pemetrexed, platinum vs placebo and pembrolizumab, pemetrexed, platinum regardless of PD-L1 expression, olomorasib in combination with pembrolizumab was found to induce intracranial activity. Early phase clinical data suggest that the combination of olomorasib and pembrolizumab can lead to significant tumor shrinkage and prolonged progression-free survival in patients with metastatic NSCLC, according to the results from the phase 1/2 LOXO-RAS-20001 trial (NCT04956640).

### RMC-6236

Sotorasib and adagrasib are limited to targeting the KRAS^G12C^ mutation, leaving a significant therapeutic gap for patients with other KRAS mutations [39]. A promising investigational compound addressing this gap is daraxonrasib (also known as RMC-6236), currently in clinical trials (NCT05379985) for the treatment of solid tumors driven by KRAS, HRAS, and NRAS mutations [39]. Unlike sotorasib and adagrasib, RMC-6236 can selectively bind to the RAS(ON) form of KRAS with mutations in KRAS^G12X^, allowing broader coverage of KRAS-MUT tumors [39]. RMC-6236 associates with cyclophilin A (CypA), a protein speculated to function in trafficking, T cell activation, and protein folding [47], with high affinity (k_d_=55.3 nmol/L) [39]. The RMC-6236–CypA complex is then able to bind to the KRAS^G12D^ (k_d_=131 nmol/L), KRAS^G12V^ (k_d_=364 nmol/L), and KRAS^WT^ (k_d_=154 nmol/L) to inhibit cell signaling [39]. Given the relatively high affinity for KRAS^WT^ receptors, researchers also examined the distribution of RMC-6236 in xenograft models. They found that the concentration of RMC-6236 was 3-7 times higher in tumor tissue than in blood, and its removal was slower than in other tissues [39]. Like adagrasib, RMC-6236 can cross the blood-brain barrier (BBB), thereby expanding the therapeutic landscape for patients with KRAS-mutant brain metastases [39]. RMC-6236 has demonstrated significant treatment response in the KRAS^G12C^ mutant mouse brain tumor xenograft model [39]. RMC 6236 monotherapy induces durable antitumor activity and frequent tumor regression across multiple KRAS^G12X^ NSCLC mouse models, including those harboring a KRAS^G12C^ mutation [39]. Due to the novelty of this compound, there is currently limited clinical trial data. While some preliminary data have been reported, additional trial results are required to draw definitive conclusions. A global phase 3 clinical trial (RASolve 301) is currently evaluating RMC-6236 in patients with advanced RAS-mutant NSCLC including those with stable brain metastases. The trial is actively recruiting patients, following its initiation in mid 2025.

## Other KRAS inhibitors with CNS activity in clinical and preclinical studies

### Fulzerasib (IBI351)

Fulzerasib (previously known as IBI351 or GFH925) is the world’s third KRAS^G12C^ inhibitor approved for NSCLC, as it received conditional approval in China in August 2024 [48]. The conditional approval was granted based on promising results of a single-arm phase 2 clinical trial with Fulzerasib monotherapy. Fulzerasib targets the cysteine residue in KRAS^G12C^, covalently and irreversibly binding to it, thereby locking the KRAS^G12C^ protein in its inactive state [49]. In a Phase 1 trial, 48 patients with NSCLC brain metastasis were enrolled. Fulzerasib demonstrated an intracranial ORR was 27.6% with a 6-month PFS of 65.0% [49]. In the phase 2 study, ORR across all patients was 49.1%, while it was 48.6% in patients with NSCLC brain metastasis [50]. The intracranial ORR was 22.6% while the intracranial disease control rate was 96.8% [50]. These early-phase results suggest CNS activity warranting further evaluation of the drug.

The KROCUS phase II study (NCT05756153) in KRAS G12C-mutant NSCLC patients evaluated the efficacy of fulzerasib (GFH925) in combination with the anti-EGFR antibody cetuximab. Of the total 27 patients, 11 (40.7%) had baseline brain metastases. Early results showed promising synergy, with an overall response rate (ORR) of 80.0% and a disease control rate (DCR) was 100% in 20 evaluable patients who had at least one post-treatment tumor assessment. Five out of seven patients (71.4%) with brain metastasis achieved partial responses [51]. The combination remained well tolerated, with grade 3 treatment-related adverse events (TRAEs) in only 18.5% [50, 51]. Importantly, efficacy in patients with brain metastases was evident, as 38.8% of NSCLC patients had brain lesions in the phase 1 cohort, and individual cases showed durable intracranial responses [52].

### Garsorasib (D-1553)

Garsorasib (D-1553) is another potent selective KRASG12C inhibitor that showed high oral bioavailability and central nervous system penetration in preclinical studies [53]. Garsorasib binds at the switch II pocket of KRASG12C in a mechanism similar to sotorasib and adagrasib to lock KRASG12C in its inactive state [53]. Patients with KRAS G12C–mutated NSCLC were administered D-1553 600 mg orally once daily, 800 mg once daily, 1200 mg once daily, 400 mg twice a day, or 600 mg twice a day in dose escalation arm [53]. In dose-expansion, all patients received 600 mg twice a day [53]. Early clinical data from NSCLC patients, including those with brain metastases, reported intracranial objective response rates (ORR) around 30-40% and disease control rates exceeding 80%, with durable responses. Among 62 NSCLC patients assessable for response at the recommended phase 2 dose, partial response was observed in 24 patients (ORR, 38.7%) and stable disease in 32 patients (DCR, 90.3%) [53]. The median progression-free survival and duration of response were 7.6 months and 6.9 months, respectively [53].

In patients with brain metastasis, phase 1/2 results showed an intracranial ORR of 17% and a DCR of 100%, but only suggested meaningful CNS penetration and stabilization of intracranial disease, although six patients were assessed and included [53]. Garsorasib (D-1553) in phase I (NCT05383898) reported overall ORR 40.5% and DCR 91.9%; in 6 brain metastasis patients. A limitation of the data is that the CNS assessment was not performed according to the RANO-BM guidelines, which makes comparisons across studies difficult [53]. In the phase 2 study results published in August 2024, patients with stable or treated brain metastasis were allowed, but were not highlighted as a separate subgroup [54]. Adagrasib showed robust intracranial efficacy in the KRYSTAL-1 trial, with an ORR of 42% and a DCR of 90% for patients with untreated brain metastases [44]. The median intracranial response duration was 12.7 months, and the median intracranial progression-free survival (PFS) was 5.4 months [44]. In comparison, Garsorasib achieved a median intracranial PFS of 7.6 to 8.2 months [54]. Its mechanistic ability to bypass efflux pumps makes it more suitable for CNS-involved NSCLC. Garsorasib has also shown an acceptable and manageable safety profile in patients with previously treated KRAS^G12C^ mutated NSCLC [54].

### BI 1823911

BI 1823911 is a next-generation, covalent, irreversible inhibitor of KRAS^G12C^ protein that binds to its inactive (GDP-loaded) state, thereby blocking downstream signaling pathways that drive tumor growth in non–small cell lung cancer (NSCLC) [55, 56]. BI 1823911 is designed by Boehringer Ingelheim [56]. Preclinical findings suggest that BI 1823911 may offer improved potency over sotorasib or adagrasib, achieving similar in vivo efficacy at lower doses (60 mg/kg vs. 100 mg/kg) and exhibiting strong anti-tumor activity across multiple KRAS^G12C^ NSCLC cell lines ([55–57] The agent is currently being evaluated in a Phase 1 clinical trial (NCT04973163) to determine its safety, tolerability, recommended phase 2 dose, and preliminary efficacy as both monotherapy and in combination with the pan-KRAS SOS1 inhibitor BI 1701963 [56, 58]. This trial includes patients with KRAS^G12C^ mutant solid tumors such as NSCLC, colorectal cancer, cholangiocarcinoma, and pancreatic adenocarcinoma, spanning both KRAS therapy naive and relapsed cases [56].

Combination approaches are a key area of investigation to enhance the depth of response and delay resistance to KRAS^G12C^ inhibitors [55] Preclinical data show that BI 1823911 demonstrates synergistic anti-tumor effects when combined with BI 1701963, as the latter shifts KRASG12C toward its GDP-bound state, the conformation that BI 1823911 targets [59]. These results supported initiation of the current combination trial. While brain metastases are a frequent and serious complication in advanced NSCLC [9, 60], and KRAS^G12C^–mutated patients are particularly prone to brain metastases [9], current reports do not specify BI 1823911’s direct efficacy in this setting. Patients with brain metastases have been included in trial cohorts, but their central nervous system activity remains under investigation. Ongoing studies of targeted agents and immunotherapies aim to overcome the challenge of limited CNS penetration in NSCLC.

### ASP2453

ASP2453 is another irreversible inhibitor of KRAS^G12C^. First characterized in 2021, ASP2453 demonstrated faster binding kinetics, higher potency, and a more extended washout period than sotorasib in a cell-free surface plasmon resonance assay. ASP2453 inhibited tumor growth in vivo in a subcutaneous xenograft mouse model using NCI-H1373 cells at as low as 5 mg/kg [61]. Tumor regression of 47% and 86% occurred, however, only at 10 mg/kg and 30 mg/kg, respectively. Despite the advantages of ASP2453 over sotorasib, it never advanced to clinical trials. While a virtual clinical trial predicted superior antitumor efficacy compared to sotorasib, ASP2453’s efficacy remains unclear in humans [61].

### MK1084

MK1084 is a next-generation, selective KRAS^G12C^ inhibitor currently under clinical development for patients with advanced NSCLC. In NSCLC. MK1084 has been evaluated as monotherapy in combination with pembrolizumab (KEYTRUDA) with or without chemotherapy (carboplatin and pemetrexed). MK1084 was assessed in combination with pembrolizumab in a clinical trial with untreated metastatic NSCLC patients who express PD-L1.

In the KANDLELIT-001 study, MK1084 demonstrated antitumor activity and a manageable safety profile in patients with advanced NSCLC, across both monotherapy and combination arms. Among 69 patients included in the trial, and with a median follow-up of 12.1 months, the overall response rate (ORR) was 77%. However, the ORR in the MK1084 monotherapy arm, which included only 21 patients with previously treated NSCLC, was 38%. Since the trial excluded patients with active CNS metastases or carcinomatous meningitis, the therapeutic outcome of MK1084 has not yet been reported in NSCLC patients with active brain metastases. Merck has initiated a phase 3 trial (NCT06345729) to evaluate the effects of MK-1084 and pembrolizumab in participants with locally advanced or metastatic NSCLC. The aims of this study are to evaluate if combining MK-1084 with pembrolizumab is superior to placebo plus pembrolizumab in terms of progression-free survival (PFS) and overall survival (OS). The trial is currently in the recruiting stage, and early results are not yet available.

### Divarasib (GDC-6036)

Divarasib (GDC-6036) is a highly selective covalent KRAS^G12C^ inhibitor. The safety of Divarasib has been established in a phase 1 GO42144 study (NCT04449874). Divarasib is 5 to 20 times as potent and up to 50 times as selective in vitro as sotorasib and adagrasib [62]. The Krascendo-170 Lung (NCT05789082), is another active trial which is a phase Ib/II, open-label study exploring the safety and efficacy of divarasib, an oral KRAS^G12C^ inhibitor, in combination with pembrolizumab [63]. In Cohort A of the trial, patients with PD-L1 expression ≥1% received divarasib plus pembrolizumab, the humanized PD-1 inhibitor. In Cohort B, patients with any level of PD-L1 expression received divarasib, pembrolizumab, platinum-based chemotherapy, and pemetrexed. Eligible participants had untreated, unresectable, or metastatic non-squamous NSCLC with a confirmed KRAS^G12C^ mutation. The dose-finding portion of Cohort A has been completed, and the trial is currently in the dose-expansion phase [63]. In patients with NSCLC (*n* = 65), divarasib treatment led to a confirmed objective response rate (ORR) of 55.6%, which increased to 59.1% at the 400 mg dose. The median duration of response (DOR) reached 18.0 months. Progression-free survival (PFS) was encouraging, with a median of 13.8 months overall and 15.3 months among those receiving 400mg [62]. To build up on these findings, two ongoing studies, the Phase 2/3 B-FAST trial (NCT03178552) and the randomized Phase III Krascendo-1 trial (NCT06497556), are evaluating divarasib further, including direct comparisons with earlier-generation KRAS^G12C^ inhibitors in pretreated populations [9].

### D3S-001

**Fig. 1:**
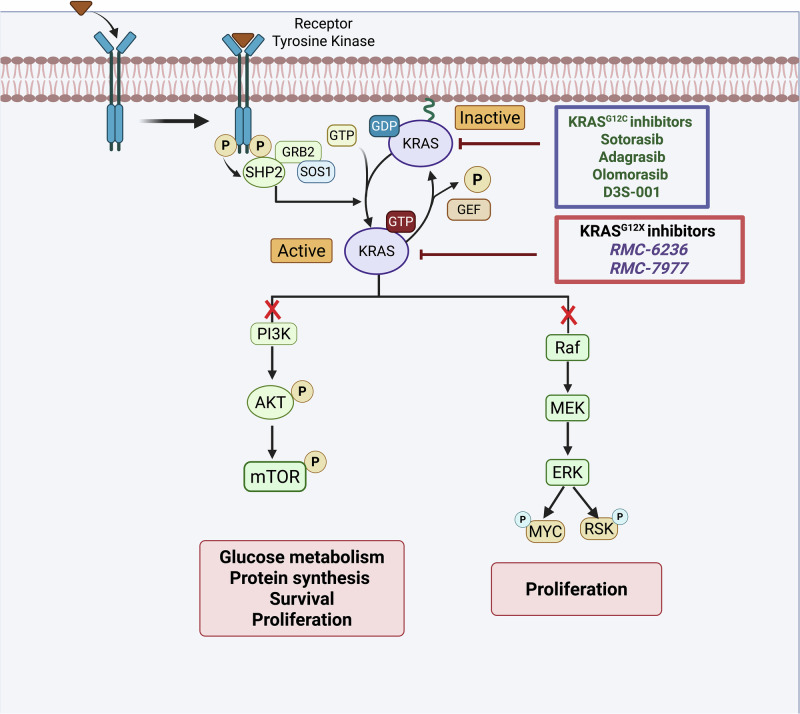
Schematic representation of the effects of KRAS inhibition on oncogenic cellular signaling. Receptor tyrosine kinase signaling promotes KRAS activation, driving downstream signaling pathways associated with tumor cellular proliferation, survival, and metabolism. The figure illustrates the mechanism by which specific KRAS (ON) and KRAS (OFF) KRAS^G12C^ inhibitors suppress downstream oncogenic signaling. Created with Biorender.com.

First-generation KRAS^G12C^ inhibitors, such as sotorasib and adagrasib, are limited by the durability of clinical responses. A new GDP-bound KRAS^G12C^ inhibitor, D3S-001, is a highly potent next-generation KRAS^G12C^ inhibitor with excellent CNS penetration and has been evaluated in preclinical models of NSCLC brain metastasis [64]. Preclinical pharmacokinetics in rats and dogs showed favorable brain exposure, enabling near-complete target engagement in the CNS at clinically relevant doses [64]. D3S-001 covalently binds to KRAS-GDP, the inactive form of KRAS, with greater potency than sotorasib or adagrasib. It outperforms earlier inhibitors in covalent potency and rapid target inhibition, resulting in significant tumor regression in brain metastasis models. In a mouse model of NSCLC brain metastasis using NCI-H1373 cells, intracranial tumor growth was reduced in mice treated with either sotorasib or adagrasib for the first 3 weeks, followed by tumor recurrence after 3 weeks of treatment [64]. In contrast, D3S-001, the new GDP-bound KRAS^G12C^ inhibitor, demonstrated rapid target engagement (TE) kinetics and sustained growth inhibition at a 30 mg/kg dose, with consistent tumor regression in the same mouse model [64]. In NCI-H358 cells, D3S-001 inhibits KRAS^G12C^ in the presence of epithelial growth factor (EGF), whereas the efficacy of either sotorasib or adagrasib wanes upon EGF administration [64]. D3S-001 has also been shown to be CNS-penetrable, with an average concentration of 7.4 nmol/L when measured in cerebrospinal fluid (CSF) [64]. Active or untreated brain metastasis is not among the exclusion criteria listed on clinicaltrials.gov. In a phase I clinical trial (NCT05410145) involving 20 patients with KRAS^G12C^ inhibitor-resistant NSCLC, D3S-001 achieved an overall response rate of 30% and a disease control rate of 80%. The drug was well-tolerated with few gastrointestinal adverse effects, such as nausea and vomiting. Low-grade liver dysfunction was the only toxicity reported. Additional results are awaited and are expected to support phase expansion of the trial. The effects of blocking KRAS activation and downstream signaling by KRAS^G12C^ inhibitors and KRASG12X inhibitors in blocking NSCLC cell proliferation and survival are summarized in Fig. 1.

## Immunotherapy with KRAS^G12C^ inhibitors

Immunotherapy remains the cornerstone of first-line treatment for patients with KRAS^G12C^ mutant NSCLC; however, outcomes for these patients remain suboptimal, especially in brain metastasis. Anti-PD-1/PD-L1 antibodies have been shown to significantly reduce tumor burden in patients with lung cancer brain metastasis [65]. Prognosis for patients with KRAS^G12C^ mutant NSCLC remains poor, despite the approval of first-generation KRAS inhibitors, which are currently limited to the second or later line of use. However, combining KRAS^G12C^ inhibitors with immunotherapy may yield a synergistic effect. Since KRASG12C inhibitors have significant immunomodulatory properties that can enhance antitumor responses, combining anti-PD (L)1 therapies with KRAS^G12C^ inhibitors may result in a synergistic effect. In a syngeneic mouse model with KRAS^G12C^, sotorasib (AMG 510) treatment, when combined with an anti-PD-1 agent, led to complete remission of tumors in 9 out of 10 mice [40]. Monotherapy with each of these agents alone results in tumor remission in only 1 out of 10 mice. Sotorasib treatment in immunocompetent NSCLC-bearing mice causes increased tumor infiltration by T cells, especially CD8 + T cells, inducing a pro-inflammatory tumor microenvironment [37, 40]. The combined efficacy of KRAS inhibitors and PD-1/PD-L1 immune checkpoint inhibitors in NSCLC brain metastasis remained poorly understood until recently. In the KRYSTAL-7 trial, MRTX849 (Adagrasib) + Pembrolizumab has been evaluated in KRAS^G12C^ mutant tumors (NCT04613596). The trial results reported that ORR 63% (32/51), DCR 84% mDOR NE (95% CI, 12.6-NE), mPFS NE (95% CI, 8.2-NE). The inclusion criteria included patients with untreated or previously treated brain metastases who did not require immediate local therapy. Patients with active brain metastasis or with any untreated brain lesions > 1.0 cm in size were excluded from the trial. Adagrasib and pembrolizumab have also been used concurrently, resulting in an improved safety profile and antitumor efficacy [57]. Anti-PD-1/PD-L1 antibodies have also been shown to significantly reduce tumor burden in patients with lung cancer brain metastasis [65]. Currently, the combined efficacy of KRAS inhibitors and PD-1/PD-L1 inhibitors in NSCLC brain metastasis is poorly understood; the Phase 2 arm of the clinical trial (NCT04613596) excludes brain metastasis. Adagrasib and pembrolizumab used concurrently had an improved safety profile and antitumor efficacy [57].

## KRAS^G12C^ targeting agent resistance mechanisms

While KRASG12C targeted therapies offer some degree of control in disease progression to many patients, the inability of these compounds to produce a durable response is an ongoing challenge. KRAS^G12C^ inhibitor resistance can be innate, acquired from a secondary mutation, or activated by resistance mediators [66]. Sotorasib and adagrasib are both OFF-state KRAS^G12C^ inhibitors. While resistance mechanisms to sotorasib and Adagrasib are best characterized, the other compounds discussed throughout this review may also suffer from similar limitations. The resistance mechanisms to sotorasib and adagrasib may be categorized into two distinct groups: genetic and non-genetic mechanisms. KRAS^G12C^ resistance may result from different kinds of genetic alterations. Secondary KRAS mutations, such as mutations in KRAS codons that include alterations at the G12C site or other KRAS sites, can interfere binding or effectiveness of KRAS^G12C^ inhibitors, ultimately leading to drug resistance [19, 67]. Mutations in KRAS occurring at other residues in addition to G12C, such as R68S and Y96C, are also characterized by increased resistance to adagrasib monotherapy. In total, 17 secondary KRAS mutations confer resistance to adagrasib and sotorasib, further strengthening the case for KRAS^G12C^ targeted therapies [68]. These mutations can occur within the KRAS switch II pocket, disrupting the binding of KRAS^G12C^ inhibitors thereby restoring the KRAS activity by either reducing GTP hydrolysis or enhancing the GDP-to-GTP exchange [19]. Loss-of-function mutations in tumor suppressors such as PTEN and NF1 can also contribute to resistance by disrupting normal signaling regulation. Additionally, co-occurring mutations in tumor suppressor genes, such as KEAP1, SMARCA4, and CDKN2A, are linked to primary resistance and poorer clinical outcomes in patients treated with KRAS^G12C^ inhibitors, like sotorasib or adagrasib [19].

Another common resistance mechanism is reactivation of MAPK pathway, where signaling is restored through alternative routes despite KRAS inhibition, allowing continued cell proliferation [66, 69]. Activation of alternative signaling pathways, such as upregulation of Hedgehog (Hh) and AXL receptor tyrosine kinase signaling, can enable tumors to avoid the KRAS inhibition [70–72]. Amplifications in receptor tyrosine kinases (RTKs) that activate KRAS are also a resistance mechanism. Activation of RTKs promotes the conversion of GDP-bound KRAS to GTP-bound KRAS. So, KRAS^G12C^ inhibitors like adagrasib and sotorasib, which are essentially KRAS(OFF) inhibitors, will lose their effectiveness during RTK stimulation [19].

Non-genetic changes like epithelial-to-mesenchymal (EMT) transition and tumor microenvironment remodeling also play key roles in resistance to KRAS-targeted therapies [73, 74]. Following treatment with transforming growth factor β, H358 cells undergo the epithelial-mesenchymal transition (EMT), leading to acquired resistance to sotorasib through sustained activation of PI3K [75].

## KRAS^G12C^ inhibitors are more efficacious when combined with targeted therapy

### Combination of Adagrasib with a cdk4/6-targeted therapy

Some clinically established putative mechanisms of Adagrasib resistance include (1) secondary mutations or amplifications in KRAS, and (2) alternative oncogenic alterations that activate the receptor tyrosine kinase–RAS signaling pathway [68]. A previous study identified that the prevalence of CDKN2A/B homozygous deletion is a common driver in lung adenocarcinoma brain metastasis [76]. CDKN2A loss leads to hyperactivation of CDK4/6 signaling. The combination of Adagrasib and a brain-penetrant CDK4/6 inhibitor abemaciclib was evaluated in NSCLC brain metastasis models driven by KRAS-G12C and *CDKN2A* loss. In a KRAS^G12C^/CDKN2A mutant SW1573 brain tumor model only the combinatorial treatment of Adagrasib and abemaciclib, but neither monotherapy, extended animal overall survival in mice. In contrast in the H2122 model of BM, both Adagrasib monotherapy and the combination treatment prolonged survival to a similar extent, while abemaciclib monotherapy was ineffective [77]. Both of these two xenograft mouse models are genetically defined by the co-occurrence of KRAS^G12C^ and CDKN2A homozygous deletion [77]. KRAS^G12C^ inhibitors like adagrasib and sotorasib can face limited effectiveness due to feedback loops and other signaling pathways that can bypass the targeted inhibition.

### Combination with a MEK-targeted therapy

Trametinib (GSK1120212), an oral, reversible and highly selective allosteric inhibitor of MEK1/MEK2 protein, demonstrated preclinical and clinical activity in KRAS-mutant NSCLC [78, 79]. Trametinib (GSK1120212), monotherapy has shown similar progression-free survival and response rates comparable to those of docetaxel in patients with previously treated KRAS-mutant NSCLC [79]. Based on preclinical and encouraging early clinical findings with trametinib in NSCLC, the combination of trametinib with KRAS^G12C^ inhibitors could be a useful therapeutic intervention in NSCLC patients for better treatment outcomes. By inhibiting MEK1/2, a protein constituent of the MAPK signaling pathway downstream of KRAS, trametinib can further suppress the MAPK signaling that KRAS activates, potentially enhancing the anti-tumor effects of the KRAS^G12C^ inhibitor, specifically Sotorasib. In a phase 1b clinical study, Ramalingam et al. reported enhanced anti-tumor activity with the combination of sotorasib and trametinib in NSCLC patients (*n* = 18 patients) previously treated with multiple lines of therapy, including KRAS^G12C^ inhibitors [80]. Out of three NSCLC patients who received prior KRAS^G12C^ therapy, two showed stable disease. Out of the HRASG12C naïve 15 patients who received other forms of therapy, the combination of Trametinib with sotorasib showed stable disease in 10 patients, 3 confirmed PR, and 1 showed Progressive disease, and 1 was reported not evaluable [80]. The study also reported that the combination of sotorasib and trametinib is safe and tolerable [80].

### Combination with a SHP2-targeted therapy

**Fig. 2:**
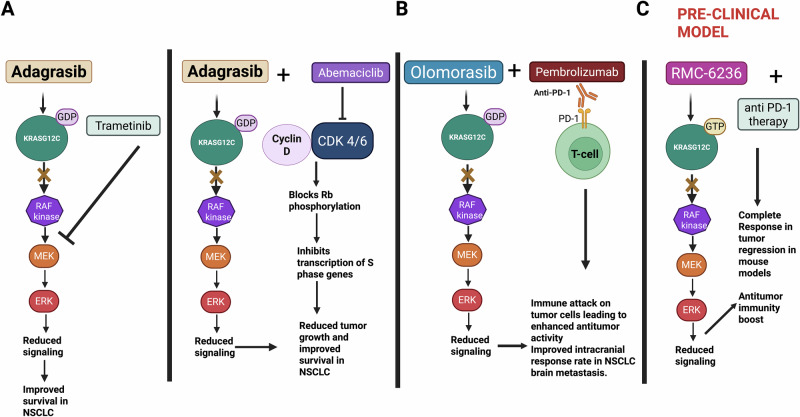
Combinatorial targeted strategies with some KRAS^G12C^ inhibitors to achieve enhanced therapeutic efficacy in non-small cell lung cancer brain metastasis. Targeted combination strategies inhibit mutant KRAS signaling and compensatory pathway components, including dual-specificity protein kinases such as MEK (**A**), cyclin D-CDK 4/6 signaling, and immune checkpoint activity (**B and C**), thereby overcoming resistance mechanisms and improving anti-tumor efficacy in KRAS^G12C^ mutant cancers. Created with Biorender.com.

Preclinical data have shown that co-inhibition of SHP2 enhances the effectiveness of KRAS^G12C^ inhibition. An ongoing clinical trial is currently evaluating the combinatorial effect of JDQ443 (a KRAS^G12C^ inhibitor) with TNO15, an SHP2 inhibitor and is currently in the final phase. The combination of SHP2 inhibitor, TNO155 and JDQ443 (a KRASG12C inhibitor) was evaluated in the KontRASt-01 (NCT04699188) phase Ib trial. Out of the 50 patients with previously treated advanced KRAS^G12C^-mutated NSCLC tumors, there were 24 patients. Out of those 24 patients, 12 patients had NSCLC and had not previously received KRA G12C inhibitor therapy; 33% of these patients showed confirmed objective responses to the combination therapy. Among 12 patients with NSCLC who had received prior KRASG12C inhibitor treatment, 33% also demonstrated confirmed responses [81]. It has been reported that combining SHP2 inhibitors (allosteric or otherwise) with KRAS^G12C^ inhibitors in vitro and in vivo shows synergy (greater tumor regression and delayed relapse) in both orthotopic and intracranial models. It has been found that RMC-4998 an inhibitor that targets RASG12C in its active, GTP-bound form, could be effective in treating KRAS mutant lung cancer in various immunocompetent mouse models. RAS pathway reactivation after RMC-4998 treatment could be delayed when the RMC-4998 treatment is combined with treatment with a SHP2 inhibitor [82]. Furthermore, combined inhibition of RAS and SHP2 in an immune-excluded model enhances tumor sensitivity to immune checkpoint blockade, resulting in effective tumor immune rejection in NSCLC [82]. This highlights the potential of combining KRAS^G12C^ inhibitors and SHP2-targeted therapy to improve treatment outcomes in NSCLC patients with brain metastasis. A schematic illustrating selected combination strategies involving KRAS^G12C^ inhibitors adagrasib, olomorasib, and RMC-6236 (daraxonrasib) is shown in Fig. 2.

## Modulation of the efficacy of radiation therapy by KRAS Inhibitors

While advances targeting KRAS have occurred in the past 10 years, conventional treatments such as surgery and radiotherapy continue to be the first line of treatment for many NSCLC patients suffering from brain metastasis. Oncogenic KRAS activating mutations confer radioresistance in tumor cells. KRAS inhibitors can restore radiosensitivity, leading to improved therapeutic outcome [83].

**Fig. 3:**
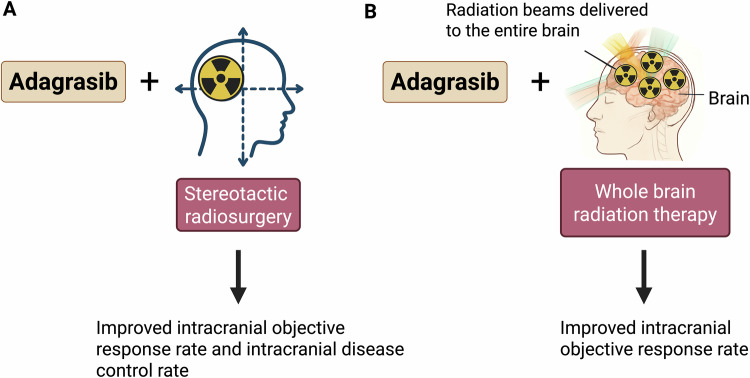
Applications of KRAS^G12C^ inhibitor Adagrasib in radiosensitization of NSCLC brain metastases. Adagrasib used in combination with either stereotactic radiosurgery (**A**) or whole-brain radiation therapy (**B**) enhances intracranial objective response rates and improves intracranial disease control, particularly with stereotactic radiosurgery. Created with Biorender.com.

Although conventional WBRT is effective, it is associated with neurocognitive side effects that diminish quality of life. More precise approaches, such as stereotactic radiosurgery (SRS), are increasingly preferred for managing brain metastases. However, patients with more than 10 metastatic brain lesions typically require WBRT. In such cases, dose de-escalation is an important consideration, and combining KRAS-targeted agents with radiation therapy may enable lower radiation doses, potentially preserving neurocognitive function and improving patient quality of life. Data suggest that sotorasib enhances the effect of radiation treatment [84, 85]. In a study using H358 and H1792 cell lines, combining sotorasib or mitogen-activated protein kinase (MEK) inhibitors with radiation therapy significantly reduced the programmed cell death ligand 1 (PD-L1) expression compared with radiation alone [85]. This is therapeutically relevant as lower PD-L1 expression in non-squamous NSCLC patients has been associated with reduced rates of synchronous brain metastasis [86]. Adagrasib is currently being evaluated in a phase 2 clinical trial (recruiting; NCT06248606) to assess its combination with stereotactic radiosurgery in patients with NSCLC brain metastases. Its use, either with stereotactic radiosurgery or whole-brain radiation therapy, has shown promising outcomes as illustrated in Fig. 3.

RMC-6236, in a phase 1 clinical trial, lacks any ongoing clinical trials to examine efficacy versus radiation therapy (RT) or in conjunction with RT. AZD4747, similarly, has no ongoing clinical trials evaluating radiation therapy in combination with AZD4747. MRTX1257, a KRAS^G12C^ inhibitor structurally related to Adagrasib, reduces the survival fraction in clonogenic assay in G12C-mutant CT26 cells when combined with radiation, in both a time and dose-dependent manner [83]. This suggests that a heterozygous G12C mutation of KRAS is not sufficient to allow a proper radio-sensitizing treatment. It has been reported that MRTX1257, a KRAS^G12C^ inhibitor radio-sensitize CT26 and LL2 cells in vitro depending on their mutation RAS mutation profile. A heterozygous G12C mutation of *KRAS* is not sufficient to for the radio sensitization induced by the combination treatment of KRAS^G12C^ inhibitor MRTX1257 and radiation. The half maximal inhibitory concentration (IC50), of MRTX1257 with mouse lung cancer LL2 WT cells, which harbor KRAS^G12C+/-^ heterozygous mutation is much higher (in the range of 2 and 10 µM) than the IC50 with 20 to 50 nM used in CT26 which are KRAS^G12C+/+^ cells [83]. MRTX1257 increases the anti-proliferative effects of RT in CT26 KRAS^G12C + /+^ tumors but not in CT26 WT tumors.

The KRYSTAL-1 phase 1b expansion cohort, which enrolled patients with active and untreated central nervous system (CNS) metastasis, showed an objective intracranial response and disease control rate of 31.6% and 84.2% respectively [87].

In immunocompetent BALB/c mice bearing CT26 xenograft tumors with a KRAS^G12C^ mutation, this combination of MRTX1257 and radiation therapy also slowed tumor growth and improved survival [83]. Interestingly, the drug pixantrone, which reduces KRAS-GTP levels demonstrated radiosensitization effect in distinct KRAS^G12C^ and KRAS^G12S^ mutated lung cancer cell lines. Studies in the A549 flank xenograft mouse model of NSCLC demonstrated that pixantrone inhibits tumor progression and prolongs survival, likely due to its radiosensitizing effect [88]. Pixantrone suppresses tumor growth by upregulating key DNA damage and senescence proteins ATM and p21, and by downregulating RAS downstream MAPK effector proteins [88]. Prochlorperazine is another drug often used during cancer treatment for its antiemetic effects. However, preclinical studies have shown that prochlorperazine acts as a radiosensitizer for a variety of KRAS mutations in tumors [89]. Prochloroperazine was found to bind and stabilize the GDP-bound state of mutant KRAS G12C, G12S, and G12V proteins. Stabilizing the GDP-bound state with prochlorperazine leads to the induction of double-strand DNA breaks and downregulation of cell cycle proteins in KRAS mutant cells, while its radiosensitizing effects are through downregulation of the Ras/Raf/MEK/ERK pathway [89].

## Conclusion

In this review, we discussed the expanding therapeutic landscape of several next-generation KRAS^G12C^ inhibitors including adagrasib, sotorasib, olomorasib, daraxonrasib (RMC-6236), and D3S-001 that have advanced into clinical trials for highly aggressive KRAS^G12C^ mutant NSCLC with brain metastases. We highlight emerging strategies that combine brain-penetrant KRAS^G12C^ inhibitors with targeted therapies, radiation therapy, or immunotherapy to overcome limitations of KRAS^G12C^ inhibitor monotherapy in NSCLC brain metastases. Depending on the signaling pathway targeted, both KRAS (ON) or KRAS (OFF) KRAS^G12C^ inhibitors can be combined with either targeted agents or PD-1 inhibitor immunotherapies to suppress parallel oncogenic signaling and enhance anti-tumor immune responses in non-small cell lung cancer. In addition, combining KRAS^G12C^ inhibitors with radiation therapy demonstrates potential for radiosensitization and improved intracranial tumor control in NSCLC brain metastasis. This strategy may enable radiation de-escalation while preserving therapeutic efficacy, particularly in patients undergoing whole-brain radiotherapy (WBRT) and in selected patients undergoing stereotactic radiosurgery (SRS). Moreover, the use of brain-permeable KRAS^G12C^ inhibitors in combination with either radiation therapy, targeted therapies, or immunotherapies represents an effective approach to overcoming adaptive therapeutic resistance in NSCLC brain metastasis. Collectively, these approaches underscore the therapeutic potential of combinatorial strategies to enhance durability the clinical benefits of KRAS^G12C^ inhibition, in NSCLC brain metastases patients.

## Data Availability

No datasets were generated or used for the research in this review article.
